# Editorial

**Published:** 1842-02-12

**Authors:** 


### THE MEDICAL EXAMINER.

### PHILADELPHIA, FEBRUARY 12, 1842.

### MISCELLANEOUS INTELLIGENCE.

*Medical Society of Richmond, Virginia.*—List of officers for 1842:—President, Robert W. Haxall, M. D.; Vice Presidents, John A. Cunningham, M. D., Socrates Maupin, M. D.; Recording Secretary, Francis P. Watkins, M. D.; Corresponding Secretary, Richard Cary Ambler, M. D.; Treasurer, James Bolton, M. D.; Librarian, George G. Minor, M. D.

*Introductory Lecture to thc Course of Chemistry in the New York University, by Professor Draper.*—A lecture of much eloquence, and a very appropriate introduction to a course of medical chemistry.

*Lecture on Heat, by Professor Draper, of the N. Y. University.*—[Published at the request of the Virginia members of the class.] A very agreeable specimen of Prof. Draper's capacity to relieve the dryness of his subject by an occasional indulgence in the glowing style in which he has on more than one occasion shown himself at home.

